# The relationship between the expression of TN and the efficiency of posterior spinal V osteotomy in patients with traumatic kyphosis

**DOI:** 10.1097/MD.0000000000009555

**Published:** 2018-02-02

**Authors:** Guohua Jiang, Yinshun Zhang, Xianjie Sun

**Affiliations:** a Department of Orthopedics, Zhejiang Rongjun Hospital, Jiaxing, Zhejiang Province; b Spine Orthopedics, the First Affiliated Hospital of Anhui Medical University, Hefei, Anhui Province, P.R. China.

**Keywords:** clinical effect, Cobb angle, complication, ELISA, posterior spinal V osteotomy, recurrence, tetranectin, traumatic kyphosis

## Abstract

**Background:**

This study was conducted with the aim to investigate the relationship between Tetranectin (TN) and efficiency of posterior spinal V osteotomy in patients with traumatic kyphosis.

**Methods:**

Ninety-two patients with traumatic kyphosis admitted in our hospital from February 2014 to June 2016 were included whose serum TN levels were examined by ELISA. Using the mean level of TN as cut-off value, patients were classified into TN high level group (group I) and TN low level group (group II). The observation indexes, including operation time, intra-operational loss of blood, Cobb angle, postoperative complications and recurrence rate of kyphosis within post-operational 6 months were recorded for comparison.

**Results:**

TN level was significantly higher in group I [(6.19 ± 0.33) μmol/L] than that in group II [(5.29 ± 0.34) μmol/L] (*P* < .05). There was no significant difference in average age, sex, lesion site and average time from injury to operation between the two groups (all *P* > 0.05). Compared to group II, operation time in group I was significantly shortened (5.02 ± 1.15 VS 4.58 ± 0.53, *P* = .023), the intra-operational loss of blood decreased (2418.56 ± 362.06 VS 2235.84 ± 325.63, *P* = .013), post-operational Cobb angle decreased (11.10 ± 1.31 VS 6.93 ± 1.04, *P* = .000), and the incidence of postoperative complications (nail-breaking, rod-breaking and looseness) and recurrence rate decreased (18.8% VS 4.5%, *P* = .036; 10.4% VS 0.0%, *P* = .028).

**Conclusion:**

Serum TN level is proved to be related to the efficiency of posterior spinal V osteotomy in patients with traumatic kyphosis, and may serve as a possible indicator for clinical treatment.

## 1 Introduction

Kyphosis is one of the most common sequela of spinal fractures.^[1]^ Traumatic kyphosis as one of the most common complications caused by nonsurgical treatment or inappropriate surgical treatment can be found in any segment of the spine.^[2,3]^ Such risk factors are all prone to occurrence of traumatic kyphosis as flexion compression and distraction type, and severe burst fracture of the spine.^[4,5]^ Once the erector muscle which responsible for maintaining the upright position of the body shrinks, along with the forward of gravity line of the spine, the angulation of the fracture becomes progressively worse.^[6]^ As one of the most complex challenging posed to all spine surgeons,^[4]^ traumatic kyphosis can result in chronic and progressive intractable pain, and even spinal cord compression.^[7]^ Optimal operation method current available for traumatic kyphosis is controversial, thus needs to be determined. Specifically speaking, some scholars support the posterior osteotomy as a safe and reliable approach considering it can provide satisfactory decompression and kyphosis correction.^[3]^ On the other hand, other scholars also point out that there may be blind area for nonopen surgery if posterior decompression approach was employed from the back to the front of the spinal cord, which may result in incomplete decompression.^[8]^ In addition, some scholars believe that the anterior approach should be taken as a preferred approach, and anterior decompression and bone graft fusion is a practical option,^[9]^ while some scholars prefer combined anterior and posterior approaches.^[10]^ To this end, the consensus regarding the optimal approach in spinal surgery shall be reached.

V osteotomy is characterized by completely closure of the intervertebral, facet joint, lamina and spinous process, larger bone contact area, small range of bone cutting, little loss of blood, small trauma, short operation time as well as high bone graft fusion rate,^[11,12]^ which serves as a preferred surgical strategy. V osteotomy also known as chevron osteotomy and the proximal chevron osteotomy is a proximal v-shaped metatarsal osteotomy which was believed to have high correction ability and a low complication rate.^[13]^ To increase bone fusion of the osteotomy surface, V osteotomy between small joints, lamina, spinous process of the kyphosis, the vertebral body and the superior vertebral body can preserve most of the vertebral pedicle and 2/3 or 1/2 of the middle and lower segment of superior articular process, as well as the vertebral lamina and spinous process of the vertebral body.^[11,14]^ It is documented that proximal chevron osteotomy is a commonly used approach which is technically demanded and evident supported that proximal chevron osteotomies have similar radiographic outcomes and clinical outcomes including pain, satisfaction, and function when compared with proximal opening wedge osteotomy for hallux valgus treatment.^[15]^

Tetranectin (TN), a trimeric protein of the C type lectin family, consisting of 3 peptide chains,^[16]^ is generally produced by such cells as monocytes, neutrophils, and fibroblasts, and widely recognized in extracellular matrix, plasma, mammary epithelium, ovary epithelium, and various endocrine glands.^[17,18]^ TN is believed to be implicated in tumor cell infiltration and metastasis by strengthening protein hydrolysis and destroying the extracellular matrix of tumors.^[19]^ Specifically, TN has been identified as an important prognostic factor for many malignant tumors, including oral cancer, ovarian cancer, and bladder cancer.^[17,20,21]^ In addition, evidence suggested that TN was very much associated with the repair of myocardium.^[22,23]^ Moreover, TN was also found to be highly expressed in skeletal muscle, which was consistent with the observation of definitive detection of tPA (and TN) at the mRNA level in human muscle.^[24,25]^ However, the relationship between TN level and the efficiency of posterior spinal V osteotomy in patients with traumatic kyphosis was seldom explored. In present study, ELISA method was used to detect serum TN level in 92 patients with traumatic kyphosis in our hospital from February 2014 to June 2016, so as to explore its clinical significance and provide new sights for evaluating the clinical efficiency of posterior spinal V osteotomy.

## 2 Materials and methods

### 2.1 Object of study

A total of 92 patients with traumatic kyphosis treated in Zhejiang Rongjun Hospital from February 2014 to June 2016 were included in present study, among which there were 58 male and 34 female. The average age of included subjects is (36.8 ± 4.5) years old. Inclusion criteria for eligible patients include: All patients were confirmed with traumatic kyphosis by clinical diagnosis and CT examination; the major clinical manifestations includes back deformity or camel shaped, ache and fatigue of traumatic lesions. Exclusion criteria were as follows: patients with other spinal related diseases; patients with other chronic or acute bone related diseases; patient with malignant tumors. The research obtained approval from the ethics committee of our hospital and supervised by the ethics committee of Zhejiang Rongjun Hospital. All eligible patients signed the informed consent and had the right to know about the experiment at the same time.

### 2.2 Therapeutic method

**Figure 1:**
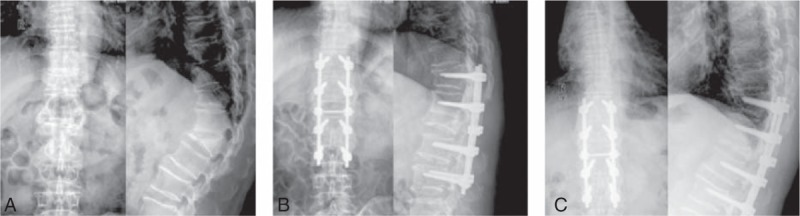
The comparison of the preoperative, intraoperative, and postoperational X-ray images in patients with traumatic kyphosis after posterior spinal V osteotomy. Note: (A) The front and lateral X-ray image before posterior spinal V osteotomy; (B) the front and lateral X-ray image of correction after posterior spinal V osteotomy; (C) X-ray showed that the fixation was firm and absence from looseness 6 months after operation.

Patients were informed to keep their prone position after anesthesia. The lesion site was examined by X-ray instrument (Shanghai Medical Instruments (Group) Ltd, Corp, AXGQ620, Shanghai, China). According to imagings, a longitudinal incision of 3 to 6 cm was made in the median posterior part of spine to fully expose the vertebral plates of the 2 to 3 vertebral bodies on the upper and lower part of the top vertebral. After X-ray set was located, both sides of the top vertebral were placed with 2 to 3 pedicle screws whose location was adjusted under X-ray, after which the vertebral plate, transverse process, and spinous process were removed and part of the vertebral plate on the adjacent vertebral bodies were removed to fully relive the pressure. Then the adhesive tissues around the endorhachis were separated till the lateral side of the vertebral body along the periosteum to fully expose bilateral vertebral bodies. Take measures to stop the bleeding in the exposed vertebral bodies. Then 2 osteotomies were inserted according to preset angle with the X-ray to confirm the osteotomy angle, which was compared to the angle obtained before surgery, thus determining the V intervertebral tissues and sclerotin to be excised. Pay attention to protect the nerve root and endorhachis. The fix rod was bilaterally placed. Then adjust the operating table to press the fix rob into the notch of the pedicle screws. Then pedicle screws were fixed by nut and pressure. Then the screw was fixed for installation of connecting rod. The crushbone was placed on the fracture gap and adjacent vertebral plate. Gelatin sponge was placed in endorhachis to stop bleeding. A drainage tube was arranged before the incision was closed. Routine monitoring of somatosensory evoked potentials (Keypoint—portable evoked potential machine, DANTEC, Alpine Biomed Aps, Skovlunde, Denmark) was performed throughout the whole operation procedure (Fig. 1).

### 2.3 Observation indexes

Following indexes including operation time, loss of blood, Cobb angle, postoperative complications (nail-breaking, rod-breaking and looseness) and recurrence rate of kyphosis after operation for 6 months were recorded for further analysis.

### 2.4 ELISA to determine serum TN levels

Before operation, 5 mL of peripheral venous blood was extracted in anticoagulant tubes from each patient at the early morning after overnight fasting. The blood samples shall be centrifuged at 2000 rpm for 10 minutes for serum separation after samples were collected for 1 hour, and then preserved at −80°C. The serum TN level was measured by double-antibody sandwich enzyme-linked immunosorbent assay (ELISA) kit (Shanghai Fan Biotechnology Co., Ltd). The prefrozen 100 μL of serum samples from patients were rewarmed to room temperature before determination, and the supernatant was collected for measurement after centrifugation.

### 2.5 Statistical analysis

SPSS 21.0 (SPSS, Inc, Chicago, IL) statistical software was applied for data analysis. *χ*^2^ was used for comparisons of enumeration data. Measurement data was expressed by mean ± standard deviation, and analyzed using *t* test between groups, and comparisons among groups were performed using one-way analysis of variance (ANOVA). A bilateral *P* < .05 indicates for statistically significance.

## 3 Results

### 3.1 Traumatic kyphosis grouping based on serum TN levels

**Figure 2:**
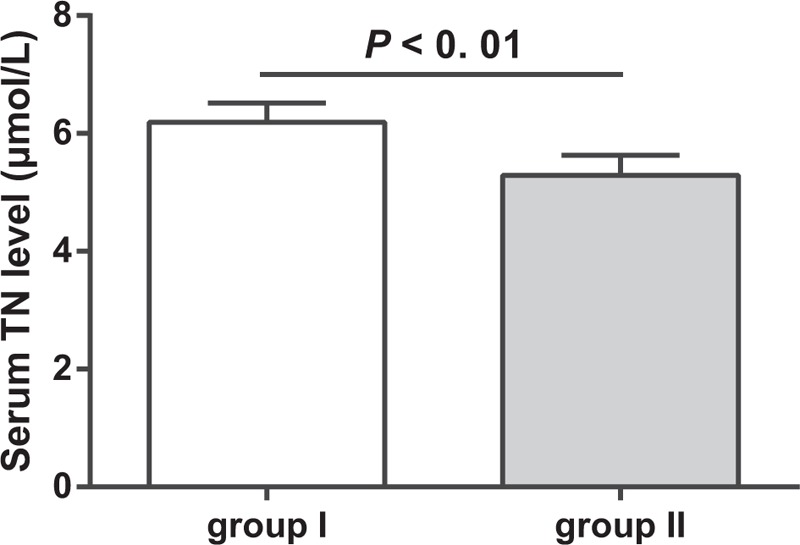
The comparison of the serum level of tetranectin in patients between groups.

ELISA showed that the average serum level of TN in all patients with traumatic kyphosis was (5.72 ± 0.56) μmol/L. Taking the average TN levels as cut-off value, patients were then grouped into group I (patients with high serum levels of TN) and group II (patients with low serum levels of TN). The serum level of TN in group I [(6.19 ± 0.33) μmol/L] is significantly higher than that in group II group [(5.29 ± 0.34) μmol/L] (*P* < .05) (Fig. 2).

### 3.2 Comparison of clinic-pathological characteristics between groups

There were 48 patients in group II, including 32 males and 16 females, with an average age of (36.1 ± 4.9) years old, among which there were 26 cases with thoracic kyphosis and 22 cases with lumbar kyphosis with an average time from operation to injury of (3.1 ± 1.1) months. Furthermore, group I had a total of 44 cases, including 26 males and 18 females, with an average age of (37.5 ± 3.8) years old. In group I, 21 patients had thoracic kyphosis and 23 patients had lumbar kyphosis; the average time from operation to injury was (2.8 ± 0.7) months. No significant difference on the average age, sex, lesion site, and average time from operation to injury between the 2 groups was detected (all *P* > .05), indicating the comparability of the 2 groups (Table 1).

**Table 1:**
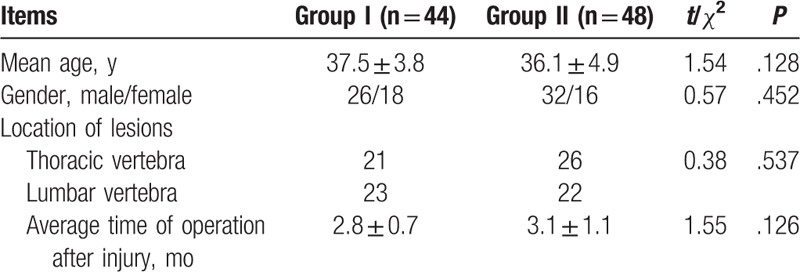
Comparison of general information between groups.

### 3.3 Comparison of operation time and the loss of blood during operation between groups

**Figure 3:**
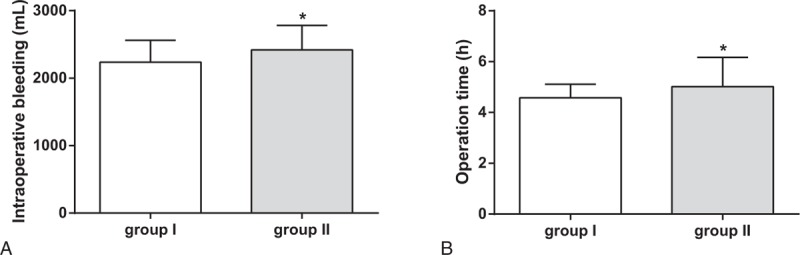
Comparisons on operation time and blood loss in patients between groups. Note: (A) Intranperative blood loss between 2 groups; (B) between 2 groups; ^∗^ compared with group I, *P* < .05.

Compared with group II, the operation time in group I was significantly shortened (5.02 ± 1.15 vs 4.58 ± 0.53, *P* = .023), amount of blood loss during operation decreased significantly (2418.56 ± 362.06 vs 2235.84 ± 325.63, *P* = .013) (Table 2, Fig. 3).

**Table 2:**
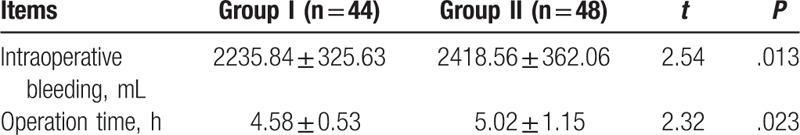
Comparison of intraoperative bleeding and operation time between groups.

### 3.4 Comparison of preoperative and postoperative Cobb angle between groups

**Figure 4:**
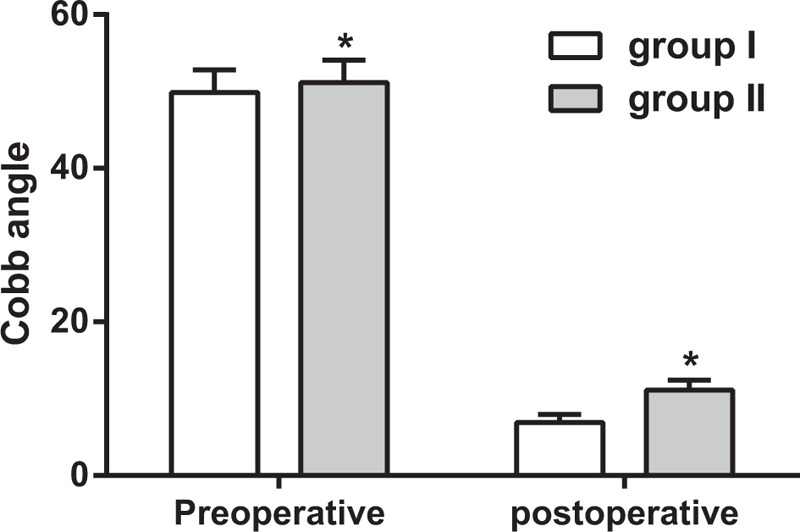
Comparisons on preoperational and postoperational Cobb angle in patients between groups. Note: ^∗^ compared with group I, *P* < .05.

As shown in Table 3, the preoperative Cobb angle was significantly larger in patients with traumatic kyphosis in group II than that in group I (51.15 ± 2.92 vs 49.89 ± 2.94, *P* = .042). Furthermore, the postoperative Cobb angle was significantly larger in group II than that in group I (11.10 ± 1.31 vs 6.93 ± 1.04, *P* = .000). However, the decrease of Cobb angle in group II was more remarkable than that in group I (*P* < .05) (Fig. 4).

**Table 3:**
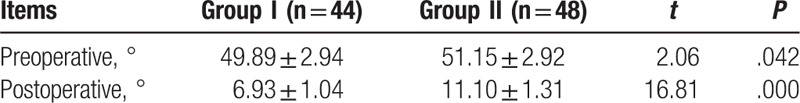
Comparison of Cobb angle between groups preoperatively and postoperatively.

### 3.5 Comparison of complications and recurrence between groups

Comparison on complications and recurrence between groups after posterior spinal V osteotomy showed that after posterior spinal V osteotomy, there were 9 cases (18.8%) had postoperative complications (nail-breaking, rod-breaking and looseness) in patients with traumatic kyphosis in group I, and 5 cases (10.4%) recurred after operation for 6 months. In parallel, as for patients with traumatic kyphosis in group II, 2 cases (4.5%) had postoperative complications, and no recurrence (0.0%) was found after operation for 6 months. The incidence of postoperative complications (nail-breaking, rod-breaking and looseness) and recurrence rate was significantly higher in patients with traumatic kyphosis in group II than those in group I (all *P* < .05) (Table 4).

**Table 4:**
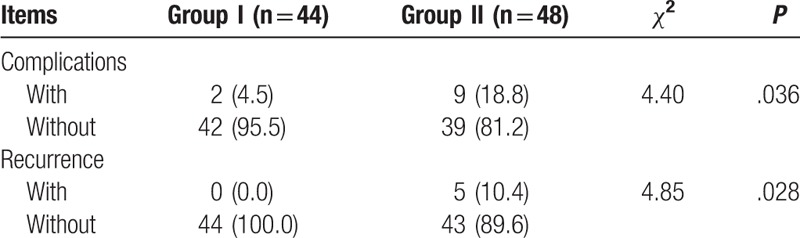
Comparison of complications and recurrence between groups [n (%)].

## 4 Discussion

TN is firstly and originally found after isolation from plasma.^[26]^ TN can specifically combine plasminogen Kringle 4 region to convert plasminogen into plasmin and promote protein hydrolysis.^[27]^ TN can also react with apolipoprotein A, tissue-type plasminogen activator, hepatocyte growth factor, and chondroitin sulfate, etc.^[17,28,29]^ The combination of TN and tissue type plasminogen activator is able to greatly enhance the activation of plasminogen. TN was found to be expressed in some endocrine cells including pituitary gland, thyroid gland, parathyroid gland, liver, pancreas, adrenal gland, and tissue epithelium and extracellular matrix.^[18,28]^ TN, rarely expressed in normal cells, is concentrated in the cytoplasm and surrounding stroma of malignant tumors, especially in the infiltrating tumor cells adjacent to normal tissues.^[19,20]^ The presentation of TN may more pronounced as the tumor progresses, suggesting that TN may implicate in tumor progression and development. In addition, TN was also found to play an important role in the development of bone, muscle, and blood vessels,^[23]^ indicating a closely association between TN and early healing of bone fracture.^[30,31]^ Meanwhile, traumatic kyphosis poses a severe healthy issue for patients,^[32]^ which may result in back pain and even severe neurological impairment or paraplegia.^[33]^ Nevertheless, the etiology of this disease remains unknown. Based on the current findings and previous research results, we therefore speculated that expression level of TN may associate with efficiency of posterior spinal V osteotomy in patients with traumatic kyphosis.

TN has long been reported to be implicated in the differentiation and maturation of bone and muscle cells, bone formation and mineralization, tissue remodeling and fracture healing.^[34]^ The implication of TN in these physiological processes may related with plasminogen in the plasminogen activator system in some extend. TN may bind to and activate the Kringle 4 region of plasminogen in a low calcium environment to break into plasmin. Proteolytic activity is extremely important in bone formation and the most important activator of MMPs zymogen is plasmin in plasminogen activator system. Study have reported that mice with gene deficiency of matrix metalloproteinase 9/gelatinase B and gelatinase B membrane type 1 matrix metalloproteinase (MTl-MMP) showed abnormalities in the process of vascularization and ossification of bone growth plates, and eventually resulted in bone development defects.^[23]^ Above results may indicate that TN may play an important role in the entire process of bone formation and bone development through the protein dissolution system.

Furthermore, decrease degree Cobb angle in patients with high serum level of TN was significantly larger than that in patient with low serum level of TN, which suggested that the high serum level of TN may indicate for a better recovery environment for patients with traumatic kyphosis. Cobb angle, which was named after the orthopedic surgeon John Robert Cobb in American, was originally used to measure coronal plane deformity on plane radiographs during the classification of scoliosis.^[35,36]^ Subsequently, it has been adopted to classify sagittal plane deformity, especially in the setting of traumatic thoracolumbar spine fractures.^[36]^ Additionally, comparison on complications (nail-breaking, rod-breaking and looseness) and recurrence rate between groups showed that the incidence of postoperative complications and recurrence rate decreased significantly in patients with traumatic kyphosis in the TN high level group than those in the TN low level group, which further support the relationship of TN expression in postoperational efficiency in patients with high level of TN. In present study, ELISA was used to determine serum levels of TN with the average serum levels of TN as the cut-off value for patient classification. Our results showed that patients with higher serum level of TN had decreased loss of blood and shorter operation time, which indicates that patients with high TN expression might have a more satisfied therapeutic efficiency. A possible explanation may be that with the increase of TN expression, corresponding abilities in differentiation and maturation of bone and muscle cells, bone formation and mineralization, tissue remodeling and fracture healing were enhanced, which in turn strengthened the postoperative recovery of patients with traumatic kyphosis.^[37]^

However, there were several limitations of this text that need to be mentioned. Firstly, due to limitation on research condition, the follow-up time in present study only reaches 6 months. In addition, the sample size of eligible patients included in present study was rather small, which can be enlarged once the conditions of hospital were improved and the research time period can be extended. In all, future studies with larger sample size and well-designed experiments were in need to confirm the relationship between TN and the efficiency of posterior spinal V osteotomy in patients with traumatic kyphosis.

In conclusion, our study confirms that serum level of TN is associated with the clinical efficiency of posterior spinal V osteotomy in patients with traumatic kyphosis, and thus may be serves as a potential indicator for patients with traumatic kyphosis after posterior spinal V osteotomy treatment.
